# Translating research evidence into practice: a report from the 2^nd^ International Conference on Maternal and Newborn Health from KLE University - Belagavi, India

**DOI:** 10.1186/s12978-018-0523-6

**Published:** 2018-06-22

**Authors:** Robert L. Goldenberg, Elizabeth M. McClure, José M. Belizán

**Affiliations:** 1 Columbia University, New York, NY USA; 2 Social, Statistical and Environmental Health Sciences, RTI International, Durham, NC USA; 3 Institute for Clinical Effectiveness (IECS-CONICET, Buenos Aires, Argentina

**Keywords:** KLE academy of higher education and research, International conference on maternal and newborn health, Pregnancy outcome research

## Abstract

The Jawaharlal Nehru Medical College (JNMC) Women’s and Children’s Health Research Unit (WCHRU) of the Karnataka Lingayat Education (KLE) Academy of Higher Education and Research Deemed-to-be-University and its collaborators convened the ‘2^nd^ International Conference on Maternal and Newborn Health -*Translating Research Evidence to Practice*’ to address the common theme of improving maternal and newborn health in low- and middle- income countries (LMIC). This supplement, including 16 manuscripts, reflects much of the research presented at the conference, including analyses of the state of knowledge, as well as completed, ongoing and planned research in these areas conducted by the WCHRU in India together with many collaborators across high-income and LMIC. The first paper reviews maternal, fetal and neonatal mortality in low-income countries, considers their causes, as well as evidence for potential interventions to reduce mortality. A second paper addresses near miss maternal mortality. Several manuscripts address the research conducted by WCHRU and their colleagues in a multi-center research network. One study examines rates of miscarriage and medically terminated pregnancy in India and the risk factors for these occurrences. Another paper addresses stillbirth and its risk factors, both in India as well as in other LMIC. Haemorrhage and preeclampsia/eclampsia, important causes of maternal mortality, stillbirth and neonatal morbidity in LMIC, are addressed in a series of papers summarizing trials of interventions to reduce improve outcomes associated with these conditions. Poor maternal and infant nutritional status, which contribute to adverse outcomes, are addressed through papers which describe a number of important studies that the WCHRU and their colleagues have conducted to attempt to improve nutritional status. Another paper describes a study to investigate causes of stillbirth and deaths among preterm births, which will utilize new techniques to investigate the infectious causes of these deaths. Finally, the supplement addresses the process for dissemination of research results to inform public policy. Together these manuscripts represent a body of research to inform interventions to reduce maternal, fetal and newborn mortality and illustrates what a dedicated research group together with institutional support can accomplish.

## Background

This *Reproductive Health* Supplement derives from presentations at a conference celebrating the work of the Jawaharlal Nehru Medical College (JNMC) Women’s and Children’s Health Research Unit (WCHRU) of the Karnataka Lingayat Education (KLE) Academy of Higher Education and Research Deemed-to-be-University and its collaborators. This ‘2^nd^ International Conference on Maternal and Newborn Health-*Translating Research Evidence to Practice*’ focuses on the efforts of the WCHRU as they move toward their goal of becoming leaders in research and advocacy related to improving maternal and child health, not only in India but in low- and middle-income countries worldwide. The primary focus of the conference is to engage various stakeholders to share completed and ongoing research and to develop a roadmap for programmatic implementation. The 16 papers in the supplement represent only a small sample of this group’s research in recent years.

### The Women’s and Children’s Health Research Unit of Jawaharlal Nehru Medical College of the KLE Academy of Higher Education and Research

The KLE Academy of Higher Education and Research Deemed-to-be-University is sponsored by the KLE Society, a charitable organization with about 13,000 members, founded in 1916. Its growth has been exponential, from a single institution in 1916 to 258 institutions in 2017, which embrace almost all disciplines of education and healthcare. Today KLE Society has over 16,000 employees and 125,000 students. One of KLE’s institutions is the Jawaharlal Nehru Medical College where the WCHRU is situated.

It is not only interesting, but also important, to understand why the WCHRU has achieved the success it has. Most important is the vision held more than 20 years ago by the group’s founders, Drs. Bhalchandra S. Kodkany and Shivaprasad S. Goudar. To support their vision, they had generous support of the KLE leadership, especially Chancellor Dr. Prabhakar Kore, a three-time Member of Parliament in India. In the late 1990’s, the KLE Society invested a substantial amount of financial resources for faculty development in the areas of medical education and perinatology through academic collaboration with University of Illinois at Chicago. The Global Network (GN) grant to KLE from the US National Institute of Health, as described later, was a direct result of that collaboration and gave the WCHRU a platform for subsequent growth and expansion.

More than many in public health at that time, Drs. Kodkany and Goudar understood the need to describe risk factors and pregnancy outcomes for specific populations. [1] Not only did they understand the need, but they also implemented systems to capture complete pregnancy-related data in a catchment area in which 10,000–15,000 women gave birth each year. Their surveillance system identified all women likely to get pregnant and therefore was able to ascertain pregnancy-related risk factors from preconception and early pregnancy. As an example, for a trial of low-dose aspirin to reduce risk of preterm birth, which required nulliparous women to be enrolled in the first trimester of pregnancy, they were able to recruit more than 70% of women prior to 10 weeks gestational age [2]. For a number of GN studies, rates of follow-up - always greater than 99% - data capture and entry within appropriate time windows, and the overall quality of data have been superb [3].

It is therefore not surprising that when funding agencies have sought sites to carry out important research projects, they often selected Drs. Kodkany and Goudar and their team. These funders include the National Institute of Child Health and Human Development (NICHD) and The Fogarty International Centre of the United States, The World Health Organization (WHO), The Bill and Melinda Gates Foundation (BMGF), The Thrasher Foundation, The UK Medical Research Council and the Department of Biotechnology, Government of India, among others.

As the opportunities have grown, the WCHRU team has expanded their catchment areas and included a number of neighboring universities. Most impressively, the JNMC WCHRU has forged a strong public-private partnership with the public-sector health care system covering more than 500 villages of the Belagavi and Bagalkot districts in India. Further, the research unit has established research collaborations with six leading medical colleges in Vijayapura, Bagalkot, Davanagere, Nagpur, Cuttack and Varanasi and has served as the coordinating center for a wide variety of research and training endeavors. They are thus teaching an ever-expanding group of Indian research scientists best research practices. As an example, when the WCHRU team at KLE was approached about a potential BMGF grant to determine cause of death in stillbirths and preterm neonates, they reached out to the JJM Medical College in Davanagere, a city located 250 km to the south of Belagavi with good clinical care but limited research experience. The KLE WCHRU team made many visits to Davanagere, worked out all the research protocols and procedures, and have assumed oversight of the project. There is little doubt that with their experience in this project, JJM Medical College in Davanagere will soon be able to participate in large international research projects on their own, with little help or oversight needed. Within India, this is a great example of both north/south and south/south collaboration.

## The NICHD global network

Because much of the work and many of the WCHRU collaborators are associated with the NICHD Global Network (GN), it is appropriate to briefly describe this network [4]. Originally funded in the year 2001 by *Eunice Kennedy Shriver* NICHD and the BMGF and continually supported since by NICHD with a number of individual studies supported by BMGF, the GN brings together seven partnerships between investigators from US institutions and investigators from low and middle-income country institutions. KLE’s WCHRU partnership is currently with Thomas Jefferson University in Philadelphia, USA.

Since 2008, each site in the GN has maintained a registry of all pregnancies and their outcomes in a defined geographic area [4]. The registry now has data on over 600,000 pregnancies. Papers in this supplement dealing with stillbirth and early pregnancy loss are derived from registry data. As important as the registry, each GN site has also defined 10 to 20 population-based clusters with about 500 deliveries per year and have randomized these clusters for important population-based studies. KLE’s WCHRU has contributed more subjects to the registry and these cluster-randomized studies than any other GN site. Examples of these include studies on the use of antenatal corticosteroids, [5] nutritional supplementation, [6] low-dose aspirin [2] and newborn resuscitation [7].

In addition, outside the GN funded research, the JNMC WCHRU is testing interventions aimed at reducing maternal and newborn mortality and morbidity from postpartum haemorrhage, preterm birth, hypertensive disorders of pregnancy and nutritional deficiencies. The outcomes of the research carried out by the unit have influenced public health policy, both nationally and globally, and have received international acclaim.

## Papers in this supplement

The papers in this supplement cover many areas - all related to the common theme of improving maternal and child health. One of the sub-themes deals with pregnancy-related mortality. The paper by Goldenberg et al. provides an overview of maternal, fetal and neonatal mortality in low-income countries, considers their causes, and explores the interventions that have been used in high-income countries to lower these mortality rates [8]. An interesting paper by Dhaded et al., using data from the GN collected by the KLE group, explores pregnancies terminating prior to 20 weeks gestation from both spontaneous miscarriage and medical termination and estimates that of 1000 pregnancies ongoing at 6 weeks, only 60% will be ongoing at 20 weeks and explores the risk factors for spontaneous miscarriages and the characteristics of women who have medical terminations [9]. Saleem et al., members of the GN from Pakistan and long-time collaborators within the GN, evaluate stillbirth rates and risk factors in seven global network sites including two in India [10]. Most importantly, they conclude that even though stillbirth rates are falling in all sites, with the current trend, the 2030 WHO goals for a stillbirth rate of 12/1000 births in every country will not be met in any GN site. Closely related to maternal mortality is serious maternal morbidity. Geller et al. summarize the research related to serious maternal morbidity throughout the world [11].

Dr. McClure, a US collaborator and the principal investigator for the GN Data Coordinating Center, in her paper, describes the study on cause of death in stillborn infants and preterm neonates that will take place in Davanagere and a site in Pakistan [12]. While there are many interesting issues raised in the paper, perhaps most important for research in India, are the introduction of minimally invasive tissue sampling as a way to achieve the goals of an autopsy without an incision and the use of multiplex polymerase chain reactions (PCR) to determine the presence of a wide variety of potentially lethal organisms in the newborns and fetuses who die. Expanding the capability of its research sites has been a goal of the GN and KLE University.

There are three additional themes for the papers in the supplement, all stemming from research conducted by KLE’s WCHRU. For example, the major killer of pregnant women worldwide and in India is post-partum hemorrhage. The KLE WCHRU group, working with their US collaborator, Dr. Derman, has been a leader in research in this area for the past 20 years. They performed some of the most important work on measuring post-partum blood loss [13] and on the use of misoprostol, an agent that can be given orally or per vagina that causes the uterus to contract and controls post-partum bleeding [14]. In this issue, Theunissen et al. describe two ongoing clinical trials using a heat stable uterotonic, Carbetocin [15]. If successful, the use of this medication will provide the most effective agent to prevent post-partum hemorrhage to be used in areas where refrigeration is not available. Also related to post-partum hemorrhage, Piaggio et al. explore methodologies to perform blood loss studies using more attainable sample sizes [16].

Another major killer of women, fetuses and newborns is preeclampsia/eclampsia. The KLE WCHRU was one of four organizations chosen by BMGF to participate in the Community-Level Interventions for Pre-eclampsia (CLIP) trial to evaluate a number of strategies related to better diagnosing and treating this disease [17]. In this issue, Katageri et al. explored the use of magnesium sulfate in the Belagavi area to reduce eclamptic seizures [18] and found that the use of this intervention was very low. Vousden et al. explore issues related to determining hypertension and shock, predominantly by accurately measuring blood pressure and emphasize the importance of accurate measurement [19]. Charanthimath et al. explore the acceptability of using lower level health workers to perform tasks such as blood pressure monitoring and administration of various medications prior to transfer in pregnant women diagnosed with various forms of hypertension [20]. Unless the diagnosis of hypertension can be made during prenatal care with transfer to a facility capable of providing appropriate care, it is unlikely that the appropriate treatments can be applied to reduce the stillbirths, asphyxia-related neonatal mortality and the maternal mortality associated with pregnancy-related hypertension. In general, they found that most people in the community, but not all physicians, approved of using lower level health workers to screen pregnant women for hypertension and provide initial treatment. The need for appropriate training was emphasized, especially by the physicians.

Still another line of KLE WCHRU research has focused on nutritional issues during pregnancy including anemia, iron deficiency and the effect of iron on development. The KLE group was one of four GN sites to participate in a major BMGF funded trial to assess the impact of preconceptional nutrition on neonatal growth, Women First, whose results will be published later this year [6]. Drs. Hambidge and Krebs review several of the issues addressed in the Women First and other trials related to nutritional supplementation to improve infant development [21]. But this represents just the latest effort to understand the relationship of maternal nutritional status and pregnancy outcome. In this issue, Masthiholi et al. present results from a study in Karnataka, India on the nutritional status of Indian pregnant women [22]. They focus on the very high prevalence of anemia, low maternal weight and food insecurity among the pregnant population. Finally, there are several papers related to iron status. Dr. Auerbach explains why iron deficiency and its related anemia is so common in countries like India, as well as why oral iron treatment is often ineffective. He makes the case for the more frequent use of intravenous iron treatment [23]. Taking a different direction, Moos et al. describe a model of iron deficiency during pregnancy in rats and the impact of low iron on rat fetal brain development [24]. They describe how maternal parenteral treatment at different times in pregnancy returns fetal brain iron stores to normal levels.

Finally, Dr. Derman considers issues related to dissemination and implementation of the results of research trials [25]. It should be clear that unless the results of research are used to improve clinical care and help to influence policies related to health care, important opportunities to improve the health of women and children are lost.

## Conclusions

In this paper, we present a very brief summary of research performed by the KLE WCHRU group and its collaborators. This effort is a great example of dedicated investigators building a research program focusing on important outcomes starting with an understanding of the need for population-based research and gradually expanding that research from observational studies to randomized controlled trials, to studies of the mechanism of disease using sophisticated technologies. As they have been building their research program, they have expanded in a number of important ways. These include adding collaborators from many different disciplines within KLE, adding collaborators from outside India including members of the GN sites, funding partners and research collaborators such as those from The University of British Columbia and the CLIP trial [17]. Finally and perhaps most impressively, the KLE WCHRU group has used their resources to expand their research into other geographic areas and to build capacity at a number of nearby universities. Instead of viewing these institutions as potential competitors, they view them as collaborators, which has increased their overall productivity. The growth and development of the KLE JNMC WCHRU over the last 20 years into a world-class research institution and their impressive accomplishments, serves as a great example and learning experience for us all.
